# Manipulation of Cellular Processes via Nucleolus Hijaking in the Course of Viral Infection in Mammals

**DOI:** 10.3390/cells10071597

**Published:** 2021-06-25

**Authors:** Olga V. Iarovaia, Elena S. Ioudinkova, Artem K. Velichko, Sergey V. Razin

**Affiliations:** Institute of Gene Biology Russian Academy of Sciences, 119334 Moscow, Russia; ioudinkova@inbox.ru (E.S.I.); velichkoak@gmail.com (A.K.V.); sergey.v.razin@usa.net (S.V.R.)

**Keywords:** nucleoli, host–virus interaction, ribosome biogenesis, apoptosis, cell cycle, snoRNAs, biocondensate

## Abstract

Due to their exceptional simplicity of organization, viruses rely on the resources, molecular mechanisms, macromolecular complexes, regulatory pathways, and functional compartments of the host cell for an effective infection process. The nucleolus plays an important role in the process of interaction between the virus and the infected cell. The interactions of viral proteins and nucleic acids with the nucleolus during the infection process are universal phenomena and have been described for almost all taxonomic groups. During infection, proteins of the nucleolus in association with viral components can be directly used for the processes of replication and transcription of viral nucleic acids and the assembly and transport of viral particles. In the course of a viral infection, the usurpation of the nucleolus functions occurs and the usurpation is accompanied by profound changes in ribosome biogenesis. Recent studies have demonstrated that the nucleolus is a multifunctional and dynamic compartment. In addition to the biogenesis of ribosomes, it is involved in regulating the cell cycle and apoptosis, responding to cellular stress, repairing DNA, and transcribing RNA polymerase II-dependent genes. A viral infection can be accompanied by targeted transport of viral proteins to the nucleolus, massive release of resident proteins of the nucleolus into the nucleoplasm and cytoplasm, the movement of non-nucleolar proteins into the nucleolar compartment, and the temporary localization of viral nucleic acids in the nucleolus. The interaction of viral and nucleolar proteins interferes with canonical and non-canonical functions of the nucleolus and results in a change in the physiology of the host cell: cell cycle arrest, intensification or arrest of ribosome biogenesis, induction or inhibition of apoptosis, and the modification of signaling cascades involved in the stress response. The nucleolus is, therefore, an important target during viral infection. In this review, we discuss the functional impact of viral proteins and nucleic acid interaction with the nucleolus during infection.

## 1. Introduction

The nucleolus is the largest nuclear compartment. The main function of the nucleolus is the synthesis of rRNA and the assembly of ribosomes [1,2,3]. In mammals, the nucleolus is assembled around tandem repeats of ribosomal DNA located in the nucleolar organizer (NOR) regions on acrocentric chromosomes. Nucleolus integrity is maintained only under conditions of active rRNA transcription [4]. Under physiological conditions, the nucleolus has a tripartite structure that consists of the fibrillar center (FC), the dense fibrillar component (DFC), and the granular component (GC). As a rule, several FCs are present in a single nucleolus and they are surrounded by DFC. Transcription of ribosomal genes occurs at the border of the FC and DFC and the processing of newly synthesized rRNA begins in the DFC and ends in the GC where ribosomes are assembled [5]. The key role in the formation of the tripartite structure of the nucleolus is played by RNA polymerase I and other factors of the transcriptional apparatus (in FC), nucleolin (in DFC), nucleophosmin (in GC), and references therein [4]. Ribosomal DNA clusters of endothermic animals consist of repeating modules containing rRNA coding regions separated by a transcribed intergenic spacer (IGS) and a non-transcribed spacer [6,7]. Each transcriptional unit is transcribed by RNA polymerase I to form 47S pre-rRNA. During processing and modification, pre-rRNA gives rise to mature 18S, 28S, and 5.8S RNA. The rRNA processing is finalized in the GC and mature rRNA becomes associated with ribosomal proteins and 5S RNA.

The modern concept of the multifunctional nucleolus [1,3,8,9] is based on proteomic data [10,11]. The proteome of the nucleolus contains more than 4500 proteins and a significant number of these proteins are not involved in the biogenesis of ribosomes. Hundreds of nucleolar proteins are involved in functional processes that are usually referred to as non-canonical functions of the nucleolus. The nucleolus contains proteins involved in controlling the cell cycle; assembling signal recognition particles; replicating and repairing DNA; regulating apoptosis and aging; detecting various kinds of stress stimuli and responding to stress; apoptosis; and aging. In addition to rRNA, snRNAs, aluRNA, tRNA (in yeast), and the RNA component of the signal recognition particle are transiently localized in the nucleolus. It is the site of additional RNA processing, including mRNA export and degradation; the maturation of uridine-rich small nuclear RNPs (U snRNPs), which form the core of the spliceosome; and biogenesis of t-RNA and microRNAs (miRNAs). Finally, the nucleolus is directly involved in maintaining the genome architecture and serves as a scaffold for the assembly of the repressive nuclear compartment [12].

Proteins diffusing in the nucleoplasm can enter membraneless nuclear compartments and either be held there or continue to move through the nuclear space. The nucleolus is a typical membraneless compartment for which its integrity is maintained by multiple weak interactions of nucleolar proteins with one another and with RNA. These promiscuous interactions that are mainly mediated by unstructured protein domains slow down the diffusion of proteins in the nucleolus [13]. The formation and maintenance of the integrity of the nucleolus, as well as of the subcompartments within the nucleolus, is best described in terms of the liquid–liquid phase separation model [4,14]. The binding of certain proteins to rRNA increases their local concentration above a threshold of phase separation. Certain amino acid motifs ensure the interaction of various nucleolar proteins with rRNA. Termed nucleolar localization signals (NoLS), these motifs are characterized by their enrichment in the positively charged amino acids [15,16]. NoLS are present in the amino acid sequences of many proteins of the nucleolar proteome. Typically, NoLS consists of 7 to 30 amino acid motifs rich in arginine and/or lysine. Despite the heterogeneity of the described NoLS, their presence in the protein is an obvious common feature of many non-nucleolar proteins transiently localized in the nucleolus. An alternative method to retain proteins in the nucleolus (including proteins that do not contain NoLS) is their interaction with chaperone molecules that possess NoLS. Nucleophosmin, a multifunctional resident protein of the nucleolus, is one such chaperone. The key role in the assembly of the nucleolus after mitosis is played by the association of nucleolin with unprocessed rRNA and nucleophosmin with partially processed rRNA. These proteins direct DFC and GC assembly around transcribed ribosomal repeats. The concept of a nucleolar protein hub, which is proposed by Emmot and Hiscox [17], suggests that resident proteins of the nucleolus, in particular nucleophosmin, can establish multiple (up to ten) contacts with partner proteins while simultaneously engaging in specific interactions with other nucleolus proteins. A liquid condensate generated by the interaction of resident nucleolar proteins can retain a significant number of client proteins. This retention (which can be considered the deposition of proteins in the nucleolus) is provided by both weak low-affinity interactions of proteins with rRNA and/or resident proteins of the nucleolus [17] and by specific strong interactions of certain proteins with resident components of the nucleolar hub.

The nucleolus is not only a multifunctional structure but also a highly dynamic structure. Assembly of the nucleolus by multiple interactions of various proteins and RNAs underlies the ability of the nucleolus to respond quickly to external and internal stimuli. Both canonical and non-canonical functions of the nucleolus are provided by the constant redistribution (the sequestration or release) of specific proteins between the nucleolus and the nucleoplasm and even the cytoplasm [17]. Data obtained using mass spectrometric analysis (SILAC) and live-cell fluorescence microscopy indicate that the protein composition of the nucleolus is very dynamic. From the point of view of flux-in and flux-out dynamics, nucleolar proteins can be conditionally divided into three groups: (i) resident proteins of the nucleolus localized mainly in the nucleolus (RNA polymerase I and fibrillarin); (ii) nucleolar multitasking proteins (nucleolin and nucleophosmin) that constantly shuttle between the nucleolus and the nucleoplasm and even the cytoplasm and in addition to participating in the biogenesis of ribosomes perform other functions; (iii) proteins that are directed to the nucleolus or leave the nucleolus exclusively in response to certain external stimuli and/or stresses, including viral infection.

Under stresses of different etiology, cardinal changes occur in the proteome of the nucleolus [18,19]. Non-nucleolar proteins are directed to the nucleolus and vice versa. Furthermore, the resident nucleolus proteins are transported to the nucleoplasm and even to the cytoplasm. The transcription of noncoding RNAs is induced from the intergenic spacers of the rDNA clusters and RNA polymerase II starts the transcription of PAPAS RNA in the direction opposite to the direction of ribosomal gene transcription [20,21]. In some cases, the transcription of ribosomal genes is repressed, which triggers the arrest of the cell cycle and/or apoptosis [22]. The need to reduce the level of ribosome biogenesis under stress is evident, as it is one of the most energy-consuming metabolic processes. Therefore, to manage stressful conditions, the cell stops transcription of ribosomal genes and mobilizes all resources to overcome the consequences of stress. Modulation of transcription of ribosomal genes is closely related to cell cycle regulation or induction of apoptosis or transformation. The nucleolus is a platform that integrates intracellular and external signals, coordinating the process of ribosome biogenesis with the physiological state of the cell.

Viruses are obligate intracellular parasites that use host cell machinery for their replication. Due to their organizational simplicity, viruses rely on cellular resources, molecular mechanisms, macromolecular complexes, functional compartments, and signaling pathways for an effective infectious process. The outcome of a viral infection can be regulated lysis of the host cell, the establishment and maintenance of persistent infection (when the cell remains alive and produces viral particles for a long period of time), and a latent infection that is accompanied by transformation and/or malignancy. The relationship between the host cell and the pathogen depends on the type of viral infection and, in each case, a unique program of interaction occurs between the pathogen and the host. Viral infection is often accompanied by targeting of viral proteins to the nucleolus, massive release of resident nucleolar proteins into the nucleoplasm and cytoplasm, and temporal localization of viral nucleic acids in the nucleolus. On the other hand, cellular proteins that are not normally present in the nucleolus can be relocated to the nucleolus and participate in the infectious process. In some cases, viruses use the nucleolus as a platform for transcription of viral genomes and post-transcriptional RNA processing. Nucleolar proteins can migrate from the nucleolus and, in association with viral proteins, participate in replicating and transcribing viral nucleic acids and assembly and the transport of viral particles in the nucleoplasm and cytoplasm. Viral proteins recruited to the nucleolus can adapt the regulatory networks that maintain cellular homeostasis for the reproduction and progression of viruses. In the latter case, the nucleolus acts as an interface in the interaction between the pathogen and the host cell. Various aspects of the interaction between the nucleolus and viruses during the infectious process are addressed in several reviews [23,24,25,26,27,28,29]. The targeting of viral proteins into the nucleolus can be accomplished using several mechanisms. Along with nuclear export and import signals, many viral proteins contain NoLS, which ensures their retention in the nucleolus. Viral proteins in the nucleolus (and the host cell’s proteins) are retained by their interaction with rRNA, rDNA, and resident proteins of the nucleolus. Some viral proteins are delivered to the nucleoli by chaperones that shuttle between the nucleoplasm and the nucleolus and interacts with viral proteins. The fact that many viral proteins contain NoLS indicates that specific mechanisms have been developed in the course of the virus’ evolution that allow it to use the nucleolus for a successful infectious process. For many years, experimental studies devoted to the study of the viral protein’s interaction with the nucleolus were focused on the identification of NoLS in viral proteins and the analysis of the dependence of the efficiency of the infectious process on the nucleolar localization of these proteins [26,29,30]. With the development of proteomics, our understanding of the range of interacting viral and cellular, including nucleolar, proteins and the functional significance of these interactions has expanded [31,32,33,34,35]. Here, we discuss different aspects of virus–host interaction assisted by the nucleolar interface.

## 2. Nucleolus as a Site of Transcription and Maturation of Viral RNAs and Assembly of Viral Particles

The interactions of viral proteins and the nucleolus are multifaceted and it is challenging to discriminate distinct functional consequences of these interactions. However, it is worth discussing separately when viral genetic materials are found in the nucleolus and the nucleolus is used as a platform for transcription and maturation of viral RNAs and as a location for the assembly and storage of virus particles.

The vast majority of RNA-containing viruses, which lack the DNA phase in the infectious cycle, replicate in the cytoplasm. However, some viruses with minus RNA strand genomes (influenza virus, Togovirus, and Borna disease virus) replicate genomic RNA in the nucleus and closely interact with the nucleolus. Early work demonstrated that the Borna disease virus uses the nucleolus as a replication site [36]. The hepatitis delta virus antigenomic RNA is transcribed in the nucleolus and the opposite polarity RNA strand is synthesized in the nucleoplasm [37]. Most of this genomic RNA appeared to colocalize with the PML nuclear bodies, providing additional evidence that HDV RNA synthesis occurs using Pol I and Pol II transcription machinery. This segregation allows the virus to make maximum use of the transcriptional apparatus and host cell nucleus compartmentalization.

During human immunodeficiency virus (HIV) infection, the virus uses the nucleolus as a “staging area” and platform for the assembly of viral RNP particles. In this case, many non-nucleolar proteins are relocated to the nucleolus and contribute to the organization of this platform along with viral proteins.

The HIV genome encodes proteins that are translated from fully spliced, partially spliced, and unspliced viral RNAs. Unspliced RNA is used as genomic RNA and mRNA for the synthesis of Gag and Gag-Pol proteins. Partially spliced RNAs are used as mRNAs for the synthesis of the Vif, Vpr, Tat, Vpu, and Env proteins, whereas fully spliced RNAs are used as mRNAs for the synthesis of Vpr, Tat, Rev, and Nef proteins. Fully spliced RNAs are stable and transported from the nucleus to the cytoplasm. In contrast, unspliced and incompletely spliced HIV RNAs are unstable and rapidly degraded in the nucleus. Sequestration of these RNAs in the nucleolus protects them from destruction. The viral Rev protein is required to transport unspliced and incompletely spliced viral RNAs to the cytoplasm and the assembly of the transport complex occurs in the nucleolus. The Rev protein possesses the nuclear localization signal and NoLS. After entering the nucleus, Rev interacts with nucleoporins Nup98 and Nup214 and exportin CRM1 and the assembled complex is transported to the nucleolus [38,39,40]. In the nucleolus, Rev multimerizes and binds to specific RRE sequences in the viral RNA [41]. The transport complex is then assembled. Export of unspliced (partially spliced) viral RNA as part of the transport complex avoids the degradation of this RNA [42].

During infection with DNA-containing adeno-associated viruses, intact viral particles are sequestered in the dormant state in the nucleolus while maintaining infectivity. During mitosis or under stressful conditions, when the nucleolus is destroyed, these viral particles acquire a chance to reach the replication sites in the nucleoplasm. Temporary sequestration in the nucleolus may be a prerequisite for the effective uncoating of the virus [43,44]. Influenza A virus double-helical ribonucleoprotein complex (vRNP) performs transcription and replication of viral genomic RNA (vRNA). Unlike most RNA viruses, vRNP formation accompanied by vRNA replication is carried out in the nucleus of virus-infected cells. All vRNP components are colocalized in the nucleolus of virus-infected cells at an early stage of infection [45].

## 3. Nucleolus as the Source of Materials for Implementation of Infectious Process

In the previous section, we discussed the usage of the nucleolus as a specialized compartment in which the viral RNA matures and is prepared for transport to the cytoplasm and virions are assembled and stored. In the context of the interaction between the virus and the host cell, this is a rather “vegetarian” scenario that is not associated with gross usurpation of the nucleolar components correlated with degradation of the nucleolus. There is an alternative strategy of interaction between the virus and the nucleolus, which involves the export of one or several components of the nucleolus into the nucleoplasm for use by the virus during the infectious process. One of the forms of resource usurpation and control of the activity of the host cell is the formation of a virus-induced microenvironment, which consists of the so-called viroplasma, viral factories, or viral replication centers (VRC) where replication and transcription of the viral genome occurs and, in some cases, also the splicing of viral RNA and the assembly of viral particles [46]. The formation of a special compartment for viral genome replication is a fairly common phenomenon in viral biology. Sequestration of the viral replicative machinery within VRC makes it possible to simultaneously solve two problems: To provide a high concentration of the necessary enzymes and to protect viral replicative factories from the host’s antiviral defense systems. Nucleolus proteins are often used as components of viral replication centers.

In cells infected with herpes viruses (HSV1 and HCMV), the structure and protein composition of the nucleolus are significantly modified during the formation of VRC [47,48]. During infection with the herpes simplex virus, the three major proteins of the nucleolus—nucleolin, nucleophosmin, and fibrillarin—as well as RPA 194, move into the VRCs where they colocalize with the viral DNA-binding protein ICP8 and participate in the replication, transcription, and the assembly of viral particles. During cytomegalovirus infection, the association of nucleolin with the viral DNA polymerase component UL44 is required for efficient DNA replication and the expression of late proteins [49]. The association of nucleolin with the viral proteins UL84 and UL44 provides, among other tasks, the maintenance of the architecture of the VRC [48,50]. Numerous nuclear proteins, including proteins of the nucleolus B23, UBF, fibrillarin, Nap140, nucleolin, POLD1, TECOFI, NOLC1, and SUMO 1 are found in the replication centers of various viruses [46,51,52]. An interesting example of the use of nucleolar proteins in the course of a viral infection is demonstrated by flaviviruses, represented by the Dengue fever virus. The capsid proteins of the virus appear in the GC of the nucleolus and displaces nucleophosmin [53]. At later stages of infection, the viral capsid protein colocalizes with nucleolin, which is involved in the assembly of mature viral particles [54,55]. Nucleolin is also present in the feline calicivirus RNA translational complex and, for efficient virus replication, the N-terminal region of nucleolin is required [56]. The WNV (West Nile virus) capsid protein binds to nucleolar RNA helicase DDX56. This complex is transported into the cytoplasm where it participates in the post-replicative assembly of viral particles [57,58]. Similarly, during infection with HIV-1, the nucleolus helicase DDX56 is released into the nucleoplasm, where it interacts with the viral protein Gag. This interaction is required for the assembly of virus particles [59].

Influenza virus (IVA) infection causes changes in the nucleolus proteome [60]. The displacement of nucleolin to the periphery of the nucleus and the redistribution of fibrillarin accompanies the accumulation of the multifunctional viral protein NS1 in the nucleolus during infection [61]. It is assumed that nucleolin provides the transport of viral ribonucleoprotein complexes and is involved in replicating viral RNA. The nucleolar protein RRP1B, which is involved in ribosome biogenesis, is translocated from the nucleolus to the nucleoplasm during infection with the influenza virus and is associated with RNA-dependent RNA polymerase, enhancing the transcription of viral RNA [62]. In addition, one of the multifunctional proteins of the nucleolus, LYAR, moves from the nucleolus to the nucleoplasm and cytoplasm, facilitating the assembly of ribonucleoprotein complexes of the influenza A virus [63].

Comparison of the proteome of nucleoli isolated from healthy cells and cells infected with adenoviruses suggest that the movement from and to the nucleolus encompasses a very wide range of proteins. As aforementioned, typical nucleolar proteins move to the viral replicative centers, while both viral proteins and multiple cellular proteins move into the nucleolus, in particular, NUP210 (a component of the pore complex), PIK 3R6 (regulatory subunit of phosphoinositide kinase), and ribosomal protein S15a [35]. Unfortunately, these observations are rather descriptive and the consequences of large-scale movements of cellular proteins during infection have not been characterized. Changes in the nucleolus proteome at the late stages of HIV-1 infection have been studied in depth. The HIV-1 Tat protein produced after integrating the viral genome triggers the transition from the latent form of infection to the productive phase associated with the expression of viral proteins and the assembly and release of viral particles. The HIV-1 Tat is found both in the nucleolus and in the nucleoplasm. Using a model for overexpression of exogenous Tat proteins in transformed Jurkat cells, it was demonstrated that Tat expression is accompanied by the movement of a wide range of cellular proteins into the nucleolus. This movement contributes to the formation of the intracellular environment and the metabolic profile of T cells that provide stable production of virions [64]. The changes in abundance of specific nucleolar proteins highlight the extensive and coordinated nucleolar reorganization in response to Tat constitutive expression. It should be mentioned, however, that the adequacy of the cell model used in the cited work raises some doubts: In natural conditions, HIV-1 does not affect transformed cells but affects normal cells. The reaction to any endogenous and exogenous stimuli of the transformed cells is obviously different from the reaction of normal cells.

Small nucleolar RNAs (snoRNAs) were first described in 1979 [65] and are currently one of the most studied classes of noncoding RNAs. SnoRNAs are 60–300 nucleotides long, highly expressed (up to 200,000 copies per cell), and deposited in the nucleolus. The most studied function of snoRNAs is a scaffolding of the protein complex mediating splicing and modification of the ribosomal and some other RNAs [66,67]. There is substantial evidence for the interaction of viruses with C/D-box small nucleolar RNAs (SNORDs). Screening using gene-trap insertional mutagenesis showed the need for SNORDs to replicate several DNA viruses (CPV and HSV2) and RNA viruses (DFV, FLU, HRV16, and RSV) [68]. Interaction between viruses and SNORDs is evidenced by the increased expression of SNORD3, SNORD44, SNORD76, and SNORD78 after viral infection with Chikungunya fever virus (CHIKV) [69]. Furthermore, significant changes in the level of SNORDs were observed in pig blood after infection with porcine reproductive and respiratory syndrome virus (PRRSV) [70]. In some cases (e.g., Moloney leukemia virus), SNORDs and other noncoding RNAs are found in virions [71]. It is assumed that these noncoding RNAs can participate in the packaging of virions. SnoRNAs are mainly localized in the nucleolus but can move into the cytosol in response to specific stimuli [72].

Cellular mRNAs contain N7-methylguanylated CAP (m7G) required for translation and export. As a rule, DNA viruses replicating in the nucleus use the host cell machinery for capping, while most RNA viruses cannot follow this path since they replicate in the cytosol. RNA viruses have developed other strategies to overcome this limitation, including “cap snatching.” Snatching the cap involves using a short fragment of the host’s RNA for the primary synthesis of viral RNA. Snatching was first detected in influenza virus infection [73] and has since been described for both nuclear and cytosolic viruses. Global RNA sequencing of human epithelial cells infected with influenza A virus (IAV H1N1/swine flu) [74] and IAV (Brisbane/59/2007) [75] showed that viruses snatch caps of numerous noncoding RNAs, as well as snoRNAs, including SNORD3, SNORD118 (U8), and SNORD13. SNORDs contain the m7G cap in the primary transcript; after export to the cytosol, the cap is hypermethylated to 2,2,7-trimethyl, which results in their nuclear re-import. Notably, some viral caps are m7G caps; that is, primary snoRNAs are snatched in the nucleus prior to their cytosolic export [76].

## 4. Changes in Transcription of rDNA and Biogenesis of Ribosomes during Viral Infection

Changes in the level of ribosomal gene transcription (up to the complete arrest) in response to changes in external factors and under stress is a universal mechanism of stress adaptation. In this case, the nucleolus is both a sensor and a coordinating center of the stress response process. Under stress conditions, when there is a need to save resources and energy, slowing down or completely stopping ribosome biogenesis is the only reasonable strategy for survival and overcoming the consequences of stress. One of the main components of the nucleolar transcriptional machinery is the TIF1A and it is a key factor linking the arrest of ribosome biogenesis with the receipt of a stress signal. Under conditions of oxidative or ribotoxic stress, c-jun N-terminal protein kinase 2 (JNK2) is activated, which triggers the process of TIF1A phosphorylation at the Thr200 position. Phosphorylation at this position disrupts the interaction of TIF1A with Pol I and SL1/TIF-1B on the ribosomal gene promoter, which in turn inhibits the initiation of transcription [77]. In addition, phosphorylation of TIF1A at Thr200 causes the transfer of this protein from the nucleolus to the nucleoplasm. Under normal conditions, the intensity of ribosome biogenesis is directly determined by the cell’s needs and, accordingly, by the intensity of cell growth. If a sufficient amount of glucose is available, TIF-1A becomes phosphorylated at positions Ser649, Ser633, and Ser199 with the participation of RSK, ERK, and mTOR, which is necessary for active transcription of ribosomal DNA [78]. Growth stimuli, such as insulin-like growth factor 1 signaling, act similarly. IGF-1 activates mTOR, which phosphorylates TIF-1A at the aforementioned positions and induces an increase in the level of rRNA transcription [79]. Lack of glucose causes arrest of rRNA transcription. In cases of an energy shortage, the AMP/ATP ratio increases and AMP-dependent protein kinase (AMPK) is activated. AMPK-dependent phosphorylation of TIF-1A at Ser635 prevents the interaction of TIF-1A with the UBF-SL1 pre-initiation complex and thus causes a block of transcription initiation [80]. Similarly, the nucleolus detects changes in the NAD (+)/NADH ratio. The eNoSC protein complex contains the nucleolar protein nucleomethylin in combination with the SIRT1 and SUV39H1 sirtuins. Changes in the NAD (+)/NADH ratio activate SIRT1 and SUV39H1, which perform deacetylation of histone H3 and its dimethylation at Lys9, respectively. Nucleomethylin targets the complex to the ribosomal genes’ promoter, causes repression of their transcription, induces a decrease in energy consumption, and restores energy balance, which protects the cell from apoptosis induced by the imbalance between energy consumption and production [81].

One could expect that a viral infection, especially one associated with transformation, should require an increase in the number of ribosomes and, accordingly, cause the enhancement of transcription of ribosomal genes. Indeed, in many cases, this is what happens and viruses can mimic some cellular mechanisms that coordinate the level of ribosome biogenesis with cellular physiology. The T antigen of the oncogenic virus SV-40 binds SL-1 in the rDNA promoter and activates rRNA transcription [82]. In addition, the SV-40 T antigen increases the level of UBF phosphorylation, which contributes to the stabilization of the UBF-SL1 complex and enhances the transcription of ribosomal genes [83]. During infection with hepatitis C virus (HCV), nonstructural protein 5A (NS5A) causes hyperphosphorylation of UBF and subsequent upregulation of ribosomal gene transcription [84]. Another viral protein of the hepatitis C virus, HCV core protein, associates with SL1 and directly interacts with the TATA-binding protein, which also causes additional activation of transcription [85]. HBx-oncoprotein of the hepatitis B virus binds to the promoter of the gene encoding TBP. An increase in TBP levels causes an increase in transcription, including the ribosomal genes [86]. In addition, HBx induces acetylation of nucleophosmin, which causes chromatin remodeling in rDNA promoters and enhances transcription [87]. Oncoproteins E6 and E7 of human papillomavirus 16 (HPV16) enhances the transcription of ribosomal genes using two different mechanisms: E7 degrades the transcription repressor of ribosomal genes, pRb, whereas E6 forms a complex with UBF1, which enhances transcription of ribosomal genes [88].

In the above-described cases, viral proteins, to a certain extent, mimic normal physiological mechanisms under proliferation induction conditions. All of these viruses have a high potential for the oncogenic transformation associated with uncontrolled proliferation and an increase in the need for protein biosynthesis and ribosome biogenesis. However, in many cases, the transcription of ribosomal genes is suppressed due to viral infection. In these cases, viral proteins target the basic machinery of RNA polymerase I-dependent transcription. For example, poliovirus inhibits the activity of RNA polymerase I by inducing the cleavage of SL-1 by a special protease and by post-translational modifications to UBF [89]. The precursors of the 3C protease of rhinovirus [90] act via the same proteolytic mechanism. Interaction of the NS1 protein of influenza virus (influenza A/Shantou/602/06 (H3N2)) with nucleolin results in hypermethylation of the rRNA promoter and repression of ribosomal gene transcription [91]. Infection with the highly pathogenic Hendra and Nipah viruses of the Henipavirus group also causes inhibition of rRNA transcription. The repression mechanism is based on the binding of the nucleolar protein Treacle by the matrix proteins of the virus. Treacle is an important mediator in the signaling chain that stops rRNA transcription in response to DNA double-strand breaks [92,93,94]. DNA double-strand breaks induce ATM-dependent phosphorylation of Treacle in complex with TOPBP1, which is necessary for its interaction with the NBS1 protein [92]. The NBS1 protein is a component of the MRE11/RAD50/NBS1 complex (MRN) that plays a critical role in the cellular response to DNA damage and the maintenance of chromosomal integrity. In turn, the interaction between Treacle and MRN is required for the transcriptional repression of rDNA in response to injury. By interacting with Treacle, viral proteins mimic the DDR response and thus induce repression of rRNA [95].

In addition to controlling the activity of RNA polymerase I, viruses implement alternative strategies to suppress ribosome biogenesis, which can be modulated at both the level of transcription and the level of rRNA processing and ribosome assembly. The herpes simplex virus (HSV-1) affects ribosome biogenesis without affecting rRNA transcription [96]. The capsid protein of the Zika virus causes the release of nucleophosmin from the nucleolus, which negatively affects the biogenesis of ribosomes since nucleophosmin is a scaffold of the nucleolar GC where ribosome assembly takes place [97]. HIV Tat protein, being overexpressed in Drosophila cells, accumulates in the nucleoli and interacts with fibrillarin and U3 snoRNA, disrupting pre-rRNA maturation.

The biological relevance of rDNA transcription suppression by viral proteins is not entirely clear. It was hypothesized that the suppression of ribosome biogenesis compromises the work of the innate antiviral immunity mechanisms and thus ensures the unhindered replication of viral DNA. In particular, it was shown that the violation of ribosome biogenesis caused the downregulation of 1392 genes, including High Mobility Group Box 2 (HMGB2), which is a chromatin-associated protein that facilitates cytoplasmic double-stranded (ds) DNA-sensing by cGAS. Furthermore, it reduced cytoplasmic HMGB2 abundance and impaired induction of interferon-beta (IFNB1) mRNA, which encodes a critical anti-proliferative, proinflammatory cytokine [98].

In the cases described here, the violation of ribosome biogenesis, on the one hand, can weaken antiviral immunity and, on the other hand, can trigger an anti-stress cellular response (see below) which protects the entire organism rather than the individual infected cells from the virus propagation. A delicate balance between the multidirectional processes triggered by a viral infection predetermines the outcome and consequences of the pathological process.

## 5. Nucleolus as a Sensor and Effector of Stress

rRNA transcription is extremely sensitive to external factors. Infection, lack of nutrients, exposure to harmful physical factors, and cytotoxic agents cause an immediate cessation of rRNA transcription and dramatic changes in the nucleolus structure: FC and DFC segregate from the GC of the nucleolus and form the so-called caps on the periphery of the nucleolus. This process is termed nucleolar stress. Nucleolar stress links stress signals to the cellular responses relative to stressful conditions. The stress response can be manifested as a change in metabolism, arrest of the cell cycle, initiation of differentiation, aging, autophagy, and cell death (when the effects of stress are irreversible). It should be noted that any disturbances in the process of ribosome biogenesis (not only arrest of rRNA transcription) can cause nucleolar stress. High-throughput proteomics of mass spectrometry-based stable isotope labeling by amino acids in cell culture (SILAC) and live-cell fluorescence microscopy of cells exposed to stress stimuli demonstrated significant changes in localization and the content of a wide range of proteins involved in the response to genotoxic and oxidative stress, as well as in rRNA transcription and processing [19].

Many signaling pathways link the maintenance of nucleolar homeostasis with the stabilization and activation of the tumor suppressor p53 [99,100,101]. p53 (often called “the guardian of the genome”) is a transcription factor that links DNA damage to cell cycle arrest (via regulation of p21 expression), induction of apoptosis (via Bax and Noxa transcription factors), or aging [102,103]. Under normal physiological conditions, a low level of p53 is maintained due to the interaction of p53 with E3-ubiquitin ligase MDM2 (HDM2), which ensures the degradation of p53 by proteasomes. Under stress, this interaction is disrupted and p53 accumulates in the cell, which activates the transcription of a range of p53-dependent genes involved in stress response. Nucleolar stress causes the release into nucleoplasm of multiple nucleolar proteins, including cyclin-dependent kinase inhibitor 2A (CDKN2A or ARF), nucleolin, nucleophosmin, nucleostemin, and ribosomal proteins L5, L11, and L23. All of these proteins compete with MDM2 and causes the release and accumulation of p53 [104,105]. The interaction of MDM2 with ribosomal proteins, due to its exceptional significance, can be modulated by many additional regulators, including PICT1 (GLTSCR2), MDMX, PICT1, PML, and NEDD8. In addition, there is reason to believe that maintaining the integrity of the nucleolus is itself necessary for the degradation of p53 under normal conditions. This requirement is due to nucleolar protein NPM1 also being able to sequester HDM2 [106]. Release of NPM1 from nucleoli can thus cause activation of p53. A specific example of this phenomenon is provided by the chain of events triggered by redox stress. Under redox stress conditions, NPM1 is S-glutathionylated, which triggers the dissociation of NPM1 from nucleolar nucleic acids [107]. NPM1 is therefore released to the nucleoplasm, where it sequesters HDM2 and causes the activation of p53.

In summary, the nucleolus takes an active part in regulating the cell cycle and apoptosis, both under normal physiological conditions and under stress and viral infection is undoubtedly a stress factor for the cell. The nucleolus appears to be an important interface that viruses utilizes to interact with the host cell and to modulate the cellular response directed at infection.

## 6. Modulation of the Cell Physiology during Viral Infection—Nucleolar Interface

The productivity of a viral infection can be increased by changing the physiology of the host cell: modulating the host cell’s antiviral immunity systems, induction or inhibition of apoptosis, induction of differentiation and aging, transformation and malignancy, and modulation of the cell cycle and stress response. Many mechanisms governing these processes under normal physiological conditions can be modified during viral infection to increase the productivity of the infectious process and virus propagation. The choice of a strategy for interaction between the virus and the cell or host organism depends on many factors; the strategy itself can radically change as the infectious process progresses. In most cases, viral infection causes cell death due to lysis, membrane destruction, or apoptosis. In some cases, cell homeostasis is practically undisturbed and the cell coexists with dormant viruses for a long time. Oncogenic viruses induce proliferation, accompanied by transformation and malignancy. Some viruses induce host cell proliferation without neoplastic cell transformation.

### 6.1. Cell Cycle Modulation

Cell cycle modulation during viral infection is a very common strategy for creating the most favorable conditions for viral reproduction, which is used by both DNA and RNA viruses, as well as retroviruses [108,109]. In most cases, viral proteins interact with pRb, cyclins, CDK, and cyclin-dependent kinase inhibitor, as well as p53, and they directly interfere with the regulation of the cell cycle [109]. DNA viruses with small genomes (small DNA tumor viruses)—polyoma virus (PV), simian virus 40 (SV40), human papilloma viruses (HPV), and adenoviruses (Ad)—infect resting cells. These viruses must activate the proliferation of the host cell to replicate their own genome. In contrast, DNA viruses with large genomes (herpes viruses) block the passage of the cell cycle, preventing entry into the S-phase and thereby ensuring the availability of factors and/or conditions inherent in strictly defined stages of the cell cycle. Delaying the cell cycle may provide certain advantages for the virus and allows additional time to complete genome replication and the assembly of viral particles [108].

In several cases, the molecular mechanisms used by viruses to modulate the cell cycle involve interactions with the nucleolus. p21Waf1/Cip1, which is the cyclin-dependent kinase (CDK) inhibitor, is the chief mediator of p53-dependent cell cycle arrest that is induced by DNA damage and other stresses [110,111]. Thus, +ssRNA alphavirus M1 induces arrest of infected cells in S-phase and subsequent apoptosis via sequestration of p21Waf1/Cip1 in the nucleolus [112].

Modulation of the cell cycle of an infected cell can also occur due to the accumulation of viral proteins in the nucleolus. Recent studies have demonstrated that the major tegument protein of human cytomegalovirus (HCMV), ppUL83 (pp65), targets the nucleolus and this targeting results in cell cycle arrest G1-G1/S by HCMV and the promotion of viral infection [113]. Localization of the viral nucleocapsid protein in the nucleolus is also typical for coronaviruses. In cells expressing the nucleocapsid protein of the avian infectious bronchitis virus (IBV), this viral protein moves into the nucleolus, which causes a redistribution of fibrillarin and nucleolin [114,115]. As a result, a delay in cytokinesis is induced and infected cells at the S/G2 border accumulate [116,117]. Multifunctional oncoprotein HBx of hepatitis B virus acts in the opposite manner. HBx enhances rDNA transcription and also induces the redistribution of the ribosomal protein RPS27a from the nucleolus to the nucleoplasm, which accelerates the progression of the cell cycle [118].

### 6.2. Induction of Apoptosis

Apoptosis can be viewed as a defense mechanism that prevents the propagation of the infection at early infection stages. At the end of the virus life cycle, apoptosis is required to effectively complete the infectious process as it promotes the egress of viral particles from the cell. Adenoviruses, baculoviruses, herpesviruses, and poxviruses infections, in most cases, are accompanied by suppression of apoptosis. Infections with Ebola, HIV-1, WNV, and Sindbis virus, in contrast, are associated with the induction of apoptosis, which results in a significant contribution to pathogenesis [119]. Apoptosis can be triggered by stimulating the host cell’s immune response or expressing viral proteins. Viral proteins can induce apoptosis by various mechanisms, including permeabilization of mitochondrial membranes, transactivation of proapoptotic genes (PUMA and Bax), and inhibition of antiapoptotic cellular proteins of the Bcl-2 family. Nucleolar stress associated with the movement of viral proteins into the nucleolus followed by accumulation of p53 is one of the primary mechanisms inducing apoptosis. The +ssRNA flavivirus family member, WNV, induces apoptosis in several cell lines, mouse brain, and skeletal muscles. Capsid proteins of this virus were shown to bind to MDM2 and mediate its sequestration in the nucleolus, thereby preventing MDM2-mediated p53 ubiquitination and hence causing an increase in the level of p53 and induction of p53-mediated apoptosis [120]. The nonstructural protein of the Schmallenberg virus not only causes the arrest of RNA polymerase II-dependent transcription but, by moving into the nucleolus, induces the release of nucleophosmin into the nucleoplasm, the subsequent degradation of the nucleoli and apoptosis [121].

In rat and human neuroprogenitor cells and rat embryonic cortical neurons, Zika virus (ZIKV) infection results in ribosomal stress characterized by the structural disruption of the nucleolus. The nucleolar presence of ZIKV capsid protein (ZIKV-C) is associated with ribosomal stress and apoptosis [122,123]. The anti-nucleolar effects are most pronounced in postmitotic neurons.

NOLC1 is a nucleolar phosphoprotein associated with both the biogenesis of the nucleolus and the cell cycle [124]. NOLC1 is an integral part of DFC and FC and it is involved in the regulation of rDNA transcription [125]. NS1 protein from H5N1 IAV induces apoptosis in A549 cells by interacting with NOLC1 and suppressing its synthesis. Notably, the interaction between NS1 and NOLC1 at an early stage of infection negatively impacts virus propagation and is part of the antiviral host defense mechanism [126]. In addition, the interaction of NS-1 with nucleolin causes hypermethylation of the ribosomal gene promoter, transcription arrest, and nucleolar stress. Accordingly, the released ribosomal proteins sequesters MDM-2 and causes the accumulation of p53.

### 6.3. Protection against Apoptosis

Normally, infected cells die as a result of activation of receptor-dependent apoptosis. The death of an infected cell prevents the propagation of the virus and is a factor of innate immunity. However, some viruses are capable of disrupting the normal regulation of the programmed cell death mechanism. Virus-induced repression of apoptosis can occur through a wide range of mechanisms. In some cases, viruses express proteins that directly interact with p53 and modulate its functions, including counteracting its proapoptotic activity. For example, HPV-16 encodes the E6 protein that, in combination with the ubiquitin ligase E6AP, forms a complex that specifically targets p53 for ubiquitin-mediated degradation [127]. HBx (hepatitis B virus protein) and adenoviral protein E1B-55K can form a complex with p53 and inhibit its DNA consensus sequence binding and transcriptional transactivator activity [128,129]. Simian virus 40 T antigene binds to the DNA-binding domain of p53 and prevents the activation of p53-dependent genes [130]. The N-protein of the coronavirus PEDV (porcine epidemic diarrhea virus) is directed to the nucleolus and, by interacting with NPM, protects nucleophosmin from proteolytic degradation by caspase-3, which increases the resistance of cells to apoptosis and increases the productivity of the virus during infection [131].

Human oncogenic viruses maintain long-term chronic infection of a single host, suggesting the necessity of manipulation of the innate immunity processes and determination of cell fate. Interacting with a wide range of nucleolar proteins, the HBx protein of hepatitis B virus simultaneously modulates proliferation processes, induces resistance to apoptosis, enhances ribosome production, and thus acts as an effector of hepatocarcinogenesis [132,133]. Infection of endothelial cells with Kaposi’s sarcoma virus induces the transcription of angiogenin (a factor that promotes angiogenesis). Angiogenin is transferred into the nucleus and nucleoli of neighboring cells, where it stimulates rDNA transcription and proliferation and inhibits apoptosis. In some cases, the host cell attempts to prevent the antiapoptotic activity of viral proteins. The nucleolar protein PICT-1 sequesters the viral antiapoptotic protein KS-Bcl-2 in the nucleolus and this prevents its access to mitochondria and, hence, suppresses the antiapoptotic activity of the virus [134]. In some cases, the interaction of viral proteins with nucleolar proteins ensures the maintenance of the latent infection. During HIV-1 virus infection, the retention of the latency state and suppression of replication is mediated by the nucleolar protein NOP2/NSUN1. Mechanistically, NOP2 associates with the HIV-1 5′LTR, interacts with HIV-1 TAR RNA by competing with the HIV-1 Tat protein, and contributes to TAR m5C methylation. RNA MTase catalytic domain (MTD) of NOP2 mediates its competition with Tat and binding with TAR. Accordingly, NOP2 suppresses HIV-1 transcription and promotes viral latency [135]. Nucleolar proteins are involved in regulating the transition from a latent to a productive infection of the Kaposi sarcoma virus. vCyclin-Cdk6 of Kaposi’s sarcoma virus phosphorylates nucleophosmin, which activates its interaction with the viral protein LANA. This activation, in turn, enhances the interaction of LANA with core histones and HDAC1 in chromatin, which ensures the repression of viral gene transcription and maintenance of the latent state of infection.

## 7. Can Viruses Corrupt the Biocondensates Constituting the Nucleolus?

In the previous sections, several examples have been presented regarding viral infection causing a massive release of nucleolar proteins into the nucleoplasm. The mechanisms for such a redistribution of nucleolar proteins remain poorly understood. One possibility is that viral proteins entering the nucleolus disturb the equilibrium in existing liquid-phase condensates, resulting in their complete or partial destruction. In this regard, it is appropriate to recall that liquid-phase condensates are dynamic structures and their integrity is maintained by a complex of multivalent interactions between their components [136,137]. This is especially true in case of biological phase condensates assembled by heterotypic interactions, such as nucleoli and other intracellular condensates [136]. In the composition of these liquid-phase condensates, it is usually possible to distinguish a limited number of resident proteins that form the condensate core and a significantly larger and variable number of client proteins that are retained in the condensate but are not important for maintaining its integrity [138]. It is known from the literature that the properties of liquid-phase protein condensates can be modulated by modifying resident proteins and attracting agents competing for interaction sites between resident proteins [137,139,140,141,142]. Competing of ribosomal proteins with nucleophosmin for the binding sites on rRNA represents a good example. During the assembly of ribosomes, the nucleophosmin bound to rRNA is displaced by ribosomal proteins, which results in the loss of the ability of mature ribosomes to reside in the granular layer of the nucleolus [136].

The most important resident proteins of the nucleolus are RNA polymerase I, which, together with other components of the transcriptional apparatus, forms FCs on the transcribed ribosomal genes; nucleolin, which forms DFC by binding to unspliced rRNA; and nucleophosmin, which forms a granular compartment by binding to assembled ribosomal particles. As already mentioned, the existence of a three-component nucleolus directly depends on rRNA transcription [4]. Targeting RNA Pol I would cause transcription arrest and the disassembly of the nucleolus. Against the background of this dramatic process, it is difficult to assess the contribution of the disintegration of FCs. When discussing the possibility of modifying the nucleolus’ liquid-phase compartments, it makes sense to focus the discussion on the DFC and the GC.

It has been reported that dipeptide repeat proteins such as poly(glycine-arginine) and poly(proline-arginine) may interact with nucleophosmin, thus preventing its interaction with other nucleolar proteins and rRNA. Preventing this interaction causes delocalization of nucleophosmin from nucleoli and concomitant disruption of the GC of the nucleolus [4]. The same scenario is likely realized during infection with the Schmallenberg virus. The viral NS protein containing intrinsically unstructured domains targeted to the nucleolus causes redistribution of nucleophosmin to the nucleoplasm [121]. Although direct interaction of the NS protein with nucleophosmin was not demonstrated experimentally, it is likely that arginine-rich stretches within the NS disordered domain compete with the interaction of nucleophosmin with resident components of the nucleolus and thus disrupts the GC liquid condensate. It would be informative to analyze the possibility of such a scenario in the case of other virus-specific proteins causing disintegration of nucleoli. The possible role of post-translational modifications of nucleolar proteins controlling their redistribution in the course of viral infection has not been properly explored. Further research is necessary to determine if and how viruses can disrupt the multiphase organization of nucleoli. One important area to explore is the modulation of nucleolar homeostasis by the accumulation of foreign proteins. Under various stress conditions, the nucleolus is frequently used for the detention of extranucleolar proteins which typically form separate phase condensates within the nucleolus (intranucleolar bodies, nucleolar aggresomes, or amyloid bodies) [14,143,144]. It will be important to find out if and how viral infection can cause the generation of such intranucleolar bodies.

## 8. Conclusions

**Figure 1:**
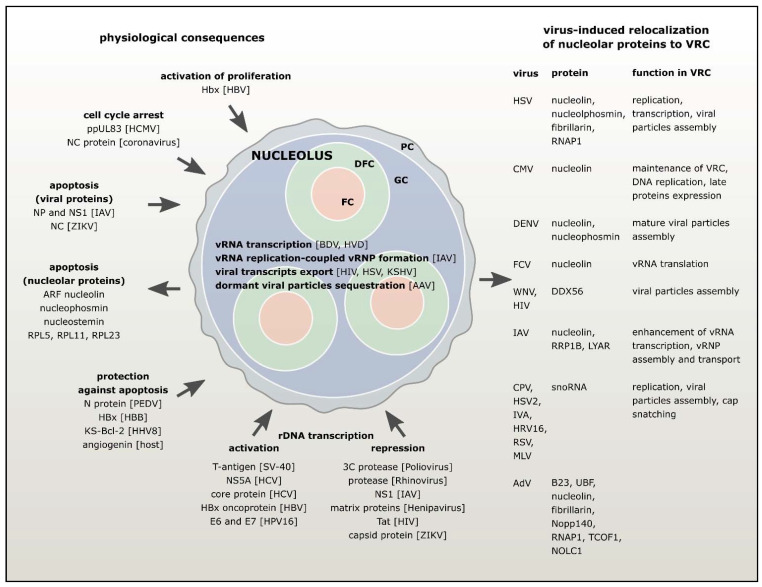
Nucleolus interface in the course of viral infection.

The virus and the host cell are in a state of constant combat, inventing an increasing number of mechanisms and counter-mechanisms to maintain an effective infection process and homeostasis of the host cell, respectively. As the central regulatory hub of the host cell and one of the main targets in the course of viral infection, the nucleolus is directly involved in the interaction of the hereditary programs of the pathogen and the host cell. In some cases, the nucleolus is used by viruses as a platform for transcription and post-transcriptional changes in viral genomes. Nucleolar proteins can migrate from the nucleolus and, in association with viral proteins, participate in replicating and transcribing viral nucleic acids and assembling and transporting viral particles in the nucleoplasm and cytoplasm. Often a viral infection is accompanied by the directed transport of viral proteins to the nucleolus. Viral proteins entering the nucleolus compete with resident nucleolar proteins for binding sites on scaffolding rRNA and disturb the equilibrium in existing liquid-phase condensates, resulting in their complete or partial destruction. Once in the nucleolus, viral proteins can directionally modulate both canonical (biogenesis of ribosomes) and non-canonical (regulation of the cell cycle and apoptosis) functions of the nucleolus. Thus, the nucleolus plays the role of an interface in the process of interaction between the pathogen and the host cell and largely determines the characteristics and the type of infection process (Figure 1).

## Data Availability

Not applicable.
